# Autohydrolysis pretreatment of Arundo donax: a comparison between microwave-assisted batch and fast heating rate flow-through reaction systems

**DOI:** 10.1186/s13068-015-0398-5

**Published:** 2015-12-21

**Authors:** Alessandro Galia, Benedetto Schiavo, Claudia Antonetti, Anna Maria Raspolli Galletti, Leonardo Interrante, Marco Lessi, Onofrio Scialdone, Maria Grazia Valenti

**Affiliations:** Dipartimento di Ingegneria Chimica Gestionale Informatica Meccanica and CIRCC, Università di Palermo, Viale delle Scienze-Ed. 6, 90128 Palermo, Italy; Dipartimento di Chimica e Chimica Industriale and CIRCC, Università di Pisa, Via G. Moruzzi, 13, Pisa, Italy

**Keywords:** Lignocellulosic biomass, Pretreatment, Liquid hot water, Autohydrolysis, Microwaves, Flow-through system

## Abstract

**Background:**

Autohydrolysis of lignocellulosic biomass in liquid hot water has been widely studied owing to its high efficiency and relatively low cost. In the perspective of industrial applications, continuous or semi-continuous processes are more interesting than batch systems. Moreover, microwave heating of pretreatment systems has been proposed to intensify the kinetics of the process. In this study, the autohydrolysis of Arundo donax was performed in pure liquid hot water using a microwave-heated batch reactor and a semi-continuous flow-through reaction system with fast heating rate at the same operating conditions with the aim of performing a systematic comparison between the two different experimental apparatuses.

**Results:**

The effect of process temperature and time, biomass to water mass to volume ratio and water flow rate on the concentration and yield of hydrolysis products was investigated. The flow-through set-up allowed us to reach biomass solubilization up to 44.5 wt% on dry basis, while the batch system stopped at 34.5 wt% suggesting that the mass transfer could be the rate-determining step in the solubilization of the constituting biopolymers. For example, in the flow-through layout, using a flow rate of 3.5 mL/min at 200 °C with 20 min of processing time, quantitative recovery of hemicellulose was obtained with limited formation of degradation products. Interestingly, higher cellulose/hemicellulose extraction ratios were found using the microwave-assisted batch reactor. FTIR analyses of the solid residues recovered after the pretreatment offered independent information on the fractions of liquefied biopolymers complementary to those derived from HPLC and UV–Vis spectroscopy.

**Conclusions:**

Collected experimental results indicated that the flow-through system can be adopted to obtain complete solubilization of the hemicellulose fraction of Arundo donax addressing the product distribution in soluble compounds towards fermentable sugars with limited formation of sugar degradation products and with limited penalty in terms of dilution of the hydrolysate solution. It was also found that microwaves can promote cellulose depolymerization and solubilization, thus allowing a more comprehensive utilization of the biomass and that infrared spectroscopy can be a useful technique to estimate the effect of the pretreatment.

## Background

Due to the exhaustible character of oil reserves, the adverse impact on climate change of CO_2_ accumulation in the atmosphere and the fluctuation of crude oil price in the world market, lignocellulosic biomass candidates as a promising alternative renewable source of fuel and value added chemicals in the biorefinery context [1]. The development of reliable technologies for the efficient conversion of biomass is a great challenge for the real exploitation of this interesting raw material.

Lignocellulosic biomass is mainly constituted by three different biopolymers, i.e. cellulose (Cell), hemicellulose (Hemi) and lignin (Lig), which are highly structured and associated with each other, in addition to other lower amounts of ashes, waxes, oils, proteins and minor compounds. The structural complexity of these materials and, in particular, their recalcitrance to hydrolysis make very difficult the scale-up of feasible and simple biomass conversion processes. For economic reasons, methodologies capable of valorizing the whole matrix are highly sought after to achieve high yields in platform chemicals and/or fuels. In this regard, polysaccharide constituents of lignocellulosic biomass, cellulose and hemicellulose, can be hydrolytically depolymerized using acid catalysts or enzymes, whereas the residual lignin solid can be used as source of phenolic and aromatic building blocks, in the perspective of a quantitative valorization of the whole matrix [2].

The efficiency of the hydrolysis process can be tuned and increased by suitable pretreatments that can be designed to maximize: (a) the yields in fermentable sugars, thus optimizing the bioethanol route; (b) the production of chemicals, such as furfural (F), hydroxymethylfurfural (HMF), levulinic acid (Lev), generated by the follow-up reactions of sugars and considered as valuable intermediates for the chemical industry, thus addressing the utilization of the raw material for the synthesis of platform chemicals [3, 4]. Several types of pretreatments, such as mechanical, physical, thermal, chemical and combinations of these, have been employed for biomass transformation, aiming at removing hemicellulose, reducing cellulose crystallinity and increasing the porosity and the surface area of the material [5]. However, all of them show technical and economic drawbacks and most of them are also energy demanding [6]. Among these pretreatment strategies, autohydrolysis by liquid hot water (LHW) has been widely studied owing to its high efficiency and relatively low cost with several experimental approaches [7–20]. In this pretreatment, only water is employed and acts as solvent as well as reagent. Hydronium ions from both water and in situ generated compounds, such as formic, acetic, levulinic, uronic and phenolic acids, catalyze the hemicellulose depolymerization, avoiding the addition of mineral acids to promote the kinetics of the process, therefore strongly decreasing corrosion problems and the need of subsequent neutralization steps. Autohydrolysis technology can be performed using both liquid water and steam, and its operating parameters can be tuned to control the kinetic severity and selectivity, thus minimizing the formation of undesired by-products. From this point of view, the way of supply the energy for the process is crucial because it mediates bond breaking between molecules during chemical action. Generally, pretreatment studies are performed in batch systems equipped with conventional heating devices, such as hot plate, heating mantles and burners, which exhibit a relatively low heat transfer rate and then require the onset of marked temperature gradients across the apparatuses. Moreover, the need of confinement of biomass under pressure increases the thermal inertia of the processing system. All these factors lead to significant sugar degradation [21].

Also microwave (MW) irradiation represents a reliable option to heat pretreatment systems, guaranteeing fast heating rates up to the process temperature with low energy inputs in comparison to those necessary with traditional heating systems [15, 17, 22–24].

MW irradiation has been widely employed in the biomass field, especially as assisted tool for the feasible catalytic conversion of lignocellulosic materials to value added compounds [25–29]. When microwave irradiation is employed, thermal energy is generated through dielectric heating and it is rapidly transferred directly into the reactor without any contact between the energy source and the reaction mixture.

On the other hand, convective or radiant heating systems are more commonly used in the thermal feeding of biomass processing. In a study of Gabhane and coworkers [30], three different heating devices, hot plate, autoclave and microwave, were tested and compared in batch reactors for the pretreatment of garden biomass under mild acid conditions in the presence of H_2_SO_4_. Effectiveness of the different modes of heating was evaluated taking into account the yield of reducing sugar, the conversion of hemicellulose into reducing sugars and the changes in the structure and in the crystallinity of the cellulose fraction of the investigated biomass. The results show that all three heating devices are useful for pretreatment; however, the efficacy of microwave is higher than that achieved with hot plate and autoclave. A maximum yield in reducing sugar of 47 % was obtained in the case of microwave heating. This value was 10 % higher than that achieved with the other two heating systems and was reached with much shorter treatment time [30]. Significant intensification effect of microwave in performing reaction has been already demonstrated by some of us in the synthesis of catalysts and in hydrogenation reactions [31, 32].

To move toward industrial-scale plants for production of bulk chemicals and fuels from biomass, efficient and sustainable systems are desirable. In this regard, a semi-continuous process in which a fixed bed of biomass is continuously treated with renewed liquid hot water represents a promising candidate, allowing high dissolution yields of the polysaccharides of the biomass with limited formation of sugar degradation products [33–35], as by adjusting the flow rate, one can control the residence time of products dissolved in the liquid stream at high temperature.

Furthermore, it is well known that the optimization of the main pretreatment process parameters is an important issue to maximize the yields towards the desirable products [20, 36].

To the best of our knowledge, no study addresses the comparison between MW and traditional heating systems with respect to their effectiveness in autohydrolysis pretreatment under comparable heating rate and also the comparison between batch and semi-continuous apparatuses was never studied before in a systematic manner. An attempt to compare the two flow regimes present in batch and semi-continuous apparatuses was done by Liu and Wyman that used a flow-through system under stopped flow condition to simulate the batch behavior [20]. However, this experimental strategy does not allow a good control of the biomass to water ratio adopted in the batch test and the comparison was carried out under stagnant conditions in the batch mode, thus amplifying the role of flow rate in the flow-through configuration.

In this paper, we have performed an experimental campaign aiming at comparing the results obtained in the autohydrolysis of Arundo donax adopting two different heating systems and flow regimes: microwave-assisted batch and fast heating rate flow-through (FT) reaction systems.

To compare the MW-based system with a more conventional heating system without uncertainties arising from differences in the chemical evolution of the biomass during the heating time, electronically controlled radiant ceramic heaters were adopted in the flow-through experimental set-up to have very short heating time of the biomass bed up to processing temperature, similarly to the case of MW irradiated reactors.

The two systems were studied under comparable conditions in terms of process temperature, treatment time and ratio between the mass of biomass and the overall volume of treatment water.

The effect of the different heating modes and flow regimes was assessed through the percentage of solubilization, the yields of oligomers, of sugars, of their degradation products and the removal of lignin.

## Results and discussions

Several set of experiments were designed and carried out to compare the performances of the two different experimental systems. The operating conditions adopted to perform the experiments are reported in each provided Table. The kinetic severity factor (KSF_s_) of the process was calculated according to the following equation [37]:

1 $$KSF_{s} = t*e^{{\left( {\frac{T - 100}{14.75}} \right)}}$$

where *t* is the cumulative treatment time [min] and *T* is the adopted operative temperature (°C).

This equation was used both for the batch system and for the semi-continuous layout. It must be precised that by this choice we are implicitly referring to the kinetic severity of the treatment for the stationary solid phase. On the other hand, the flow-through process operates batchwisely for what concerns the solid biomass, while it is an open reactor referred to the aqueous stream. For this reason, two different kinetic severity factors should be considered to correlate the performances of the process: one referred to the solid phase, defined by Eq. 1, and another one to correlate the chemical evolution of dissolved products that is dependent on the average residence time *τ* of the liquid stream inside the vessel (Eq. 2):

2 $$KSF_{l} = \tau *e^{{\left( {\frac{T - 100}{14.75}} \right)}}$$

In Eq. 2, the average residence time *τ* of the aqueous phase inside the reactor was simply estimated by the ratio between the nominal reactor free volume (30 mL) and the adopted volumetric flow rate [mL/min].

The pretreatment of lignocellulosic biomass in LHW occurs through a sequence of consecutive steps. It seems reasonably to hypothesize that hemicellulose and, in much more limited extent, amorphous cellulose domains are swollen by liquid hot water whose autoprotolysis activates the depolymerization of the polysaccharides with formation of soluble oligomers that are transferred from the solid to the aqueous phase. Once solvated, these oligomeric species can evolve undergoing depolymerization to constituent sugars that can further be converted in degradation products, such as F, HMF, Lev and formic acids (FA) [38].

According to the literature, after 20 min of treatment time, the solubilization by LHW process of biopolymers constituting Arundo donax is very poor at temperatures lower than 165 °C [39], while significant degradation of monosaccharides was observed at temperature higher than 200 °C in batch systems [40]. These data clearly highlight the role of temperature in determining the total amount of solubilized biomass as well as the product distribution. On the other hand, according to the previously mentioned description of the process at the molecular level, the hydrothermal treatment should give different distribution patterns of the products in batch or open systems.

In particular, in the case of a batch process, exposure time at high temperature is the same for both the solid and the liquid phase, while in the case of flow-through systems, changing the flow rate, the residence time of the dissolved products at high temperature can be changed independently on the processing time of the solid matrix. This means that in the open system lower concentration of degradation products can be achieved as a consequence of a shorter exposure of the dissolved sugars to high temperature.

More delicate to be discussed is the effect of the flow-dynamic regime on the yield in dissolved products. Indeed, if the rate-determining step (r.d.s.) of the dissolution process is the solid-state depolymerization of the biopolymers in the swollen matrix, similar total yields in soluble compounds should be obtained in the two different systems provided that the temperature and the treatment time of the matrix are the same. Differently, if the r.d.s. is the mass transfer of oligomers generated in the solid matrix to the aqueous phase, flow-through system should lead to faster dissolution rate owing to the better hydrodynamic regime that should induce higher values of mass transfer coefficients.

### Influence of the process temperature

A set of experiments was carried out to compare the performances of the process as a function of the operating temperature in the two different experimental apparatuses. The comparison was performed at 180 and 200 °C, using operating conditions reported in Tables 1 and 2. Experiments were performed keeping fixed in the two different systems the total treatment time, the nominal biomass concentration in water and the kinetic severity referred to the stationary biomass KSF_S_. In spite of this, different values of monosaccharide concentrations were obtained with the two different experimental set-ups (compare Exp. 1FT with 1MW and 2FT with 2MW in Table 1) presumably as a consequence of the different kinetic severity for the liquid phase (KSF_L_) that was lower for the FT system. This could lead to a lower depolymerization of solubilized hemicellulose oligomers that led to lower concentration of xylose (Xyl), arabinose (Arab) and glucose (Glu) detected in the liquid solutions drained from this system.

**Table 1:** Comparison between microwave-assisted batch and fast heating rate flow-through reaction systems: effect of operative temperature on the product concentrations in the liquid hydrolysate

Reaction conditions								Monomer conc.			Oligomer/polymer conc.			Degradation product conc.				
Exp.	T ( °C)	t (min)	τ (min)	Q (mL/min)	M_b_/V_w_ (g/L)	Log(KSF_S_)	Log(KSF_L_)	Glu (g/L)	Xyl (g/L)	Arab (g/L)	ASL (g/L)	Olig (Cell) (g/L)	Olig(Hemi) (g/L)	Acet (g/L)	Lev (g/L)	HMF (g/L)	F (g/L)	FA (g/L)
1FT	180	20	4.3	7.0	50	3.66	2.99	0.1	0.3	0.3	3.6	0.6	7.1	0.2	0.0	0.0	0.0	0.1
2FT	200	20	4.3	7.0	50	4.25	3.58	0.2	1.8	0.4	3.8	0.6	9.3	0.2	0.0	0.0	0.1	0.0
1MW	180	20	–	–	50	3.66	3.66	0.4	1.1	0.9	2.2	2.4	4.9	0.0	0.2	0.0	0.0	0.0
2MW	200	20	–	–	50	4.25	4.25	0.9	4.8	1.6	2.2	1.1	2.3	0.2	0.7	<0.1	0.0	0.0

*τ* average residence time, *Q* water flow rate, *M*_*b*_ */V*_*w*_ biomass to water ratio (w/vol), *KSF*_*S*_ kinetic severity factor for the solid phase, *KSF*_*L*_ kinetic severity factor for the liquid phase, *Glu* glucose, *Xyl* xylose, *Arab* arabinose, *ASL* acid-soluble lignin, *Cell* cellulose, *Hemi* hemicellulose, *Acet* acetic acid, *Lev* levulinic acid, *HMF* hydroxymethylfurfural, *F* furfural, *FA* formic acid

**Table 2:** Comparison between microwave-assisted batch and fast heating rate flow-through reaction systems: effect of operative temperature on the product yields (% w/w)

Reaction conditions								X_tot_ (%)	Monomer yield			Oligomer/polymer yield			Degradation product yield				
Exp.	T (°C)	t (min)	τ (min)	Q (mL/min)	M_b_/V_w_ (g/L)	Log (KSF_S_)	Log (KSF_L_)		Y_Glu_ (%)	Y_Xyl_ (%)	Y_Arab_ (%)	Y_ASL_ (%)	Y_olig(Cell)_ (%)	Y_olig(Hemi)_ (%)	Y_Acet_ (%)	Y_Lev_ (%)	Y_HMF_ (%)	Y_F_ (%)	Y_FA_ (%)
1FT	180	20	4.3	7.0	50	3.66	2.99	28.5	0.2	0.6	0.6	7.7	1.3	15.2	0.4	0.0	0.0	0.0	0.1
2FT	200	20	4.3	7.0	50	4.25	3.58	40.3	0.4	4.6	0.9	9.7	1.7	24.0	0.6	0.0	0.0	0.2	0.0
1MW	180	20	–	–	50	3.66	3.66	29.0	0.7	2.2	1.7	4.4	4.8	9.8	0.0	0.4	0.0	0.0	0.0
2MW	200	20	–	–	50	4.25	4.25	34.0	1.8	9.8	3.2	4.5	2.3	4.5	0.5	1.4	0.0	0.0	0.0

*τ* average residence time, *Q* water flow rate, *M*_*b*_ */V*_*w*_ biomass to water ratio (w/vol), *KSF*_*S*_ kinetic severity factor for the solid phase, *KSF*_*L*_ kinetic severity factor for the liquid phase, *X*_*tot*_ total solubilized fraction of the initial biomass calculated by Eq. 8, *Glu* glucose, *Xyl* xylose, *Arab* arabinose, *ASL* acid-soluble lignin, *Cell* cellulose, *Hemi* hemicellulose, *Acet* acetic acid, *Lev* levulinic acid, *HMF* hydroxymethilfurfural, *F* furfural, *FA* formic acid

To obtain further indication, one can consider the combined yields in solubilized C5 monosaccharides and hemicellulose oligomers (Table 2). Significantly higher values of this parameter were obtained in the open system with respect to the batch reactor (Table 2) corresponding to higher fractions of hemicellulose extracted from the loaded biomass (Table 3), as a probable result of the more efficacy mass transfer from the solid matrix to the aqueous phase. Moreover, negligible amounts of degradation products were found at both 180 and 200 °C in the hydrolysate recovered from the FT system. These data are coherent with the possibility that oligomers undergo limited depolymerization in the fixed bed reactor and it is a clear indication that the rate of dissolution of the biopolymers from the solid matrix is dependent on the flow regime being faster in the open system. In particular, quantitative recovery of hemicellulose fraction was obtained in the FT system at 200 °C with an average residence time of the liquid in the reactor of 4.3 min (Exp. 2FT in Table 3). These results strongly support the hypothesis that the dissolution process is mass transfer controlled as proposed by Liu and Wyman [20].

**Table 3:** Cellulose and hemicellulose extraction in experiments carried out at different reaction temperature

Experiment	Cellulose extraction (% w/w)	Hemicellulose extraction (% w/w)	Extracted Cellulose/Hemicellulose
1FT	4	56	0.07
2FT	6	~100	0.06
1MW	16	44	0.37
2MW	16	55	0.30

For the calculations of the reported values, see the section “Methods”

As expected, when the temperature was increased from 180 to 200 °C, both the concentration and the fraction of solubilized biomass increased in both systems (from 28.5 to 40.3 % in the flow-through and from 29.0 to 34.0 % in the batch system as reported in Table 2). At all investigated temperatures, in the continuous system, oligomer yields were in strong excess with respect to monosaccharide yields. In the MW-assisted batch process at 180 °C, oligomer yields were still higher than yields of monosaccharides even if values were closer each other. When the kinetic severity was increased performing the treatment at 200 °C (Exp. 2MW in Table 2), a clear inversion between the relative yields of sugars and oligomers was observed only for the batch system, xylose being the product with the highest estimated yields (9.8 % compared with 4.5 % of hemicellulose oligomers). The different behavior of the two systems when the temperature was increased can be again attributed to their different flow-dynamic regimes and severity factors for the liquid phase. Hydrolysis occurred more intensely in the batch reactor, as indicated by the higher amount of monomeric sugars detected in the hydrolysate, owing to the decrease of pH induced by accumulation of acidic compounds derived from biopolymer transformations [41]. In the case of the FT layout, the aqueous stream conveys out of the reactor the extracted oligomers as well as the aforementioned organic acids, thus decreasing the local concentration of in situ generated acid catalysts. Interestingly, even if the amount of monomeric sugars is always higher in the batch-derived hydrolysate, at the highest investigated temperature, the total amount of solubilized biomass is higher in the flow-through process (40.3 % compared to that found for the batch system that was 34.0 % as reported in Table 2). This indicates that in the FT layout the solubilized oligomers cannot be completely depolymerized to their monomeric constituents during their residual residence time inside the semi-continuous reactor.

An interesting aspect is related to the presence in the hydrolysate of species coming from the cellulose dissolution, i.e. glucose, gluco-oligomers and levulinic acid [38, 41]. The concentration of these species as well as the mass ratio between the solubilized cellulose and hemicellulose are higher for the MW-batch process with respect to the FT (Table 3).

This evidence seems to suggest a specific action of the microwaves on the cellulosic fraction of the biomass. Hydrogen bonding is a critical factor in polysaccharide structure and it is well known that the depolymerization of cellulose depends strongly on the structure of the hydrogen bond network [42]. In particular, the structure of cellulose involves intersheet, interchain and intrachain hydrogen bonds which impart rigidity and stability to the cellulose structure but which can be broken at elevated temperatures [43]. More specifically, it is reported in the literature [44] that when microwave irradiation has been employed for the depolymerization of microcrystalline cellulose consisting of both amorphous (13 %) and crystalline (87 %) regions, at temperature below 180 °C the –CH_2_OH groups of cellulose are hindered from interacting with microwave, being strongly involved in the hydrogen bonding within both the amorphous and crystalline regions. On the other hand, above the softening temperature (180 °C), these functional groups could be involved in a localized rotation in the presence of microwave, acting similarly as “molecular radiators” allowing the transfer of microwave energy to their surrounding environment. On the basis of these elements, taking into account that the employed temperature is equal or higher to 180 °C, it is possible to explain the higher depolymerization of cellulose fraction when microwave irradiation was employed, suggesting a specific relationship between cellulose fraction decomposition and microwave activation of the –CH_2_OH pendant groups. This hypothesis results in agreement with literature data where the beneficial effect of microwave toward cellulose hydrolysis has been reported, especially in the presence of catalysts [45, 46].

Apart from carbohydrate-derived compounds, also acid-soluble lignin (ASL) fractions were detected in the liquid solutions obtained after pretreatments. For the batch process, the yield in ASL does not seem to be significantly affected by a temperature increase remaining close to 4.5 % (Exp. 1MW and 2MW in Table 2). Conversely, the temperature affected the ASL yield in FT process since its value increased from 7.7 to 9.7 % increasing the temperature from 180 to 200 °C (Exp. 1FT and 2FT in Table 2). Furthermore, higher ASL yields were obtained with the FT process. A possible explanation of these different results can be proposed considering the behavior of ASL. It is generally assumed that portion of lignin chemically crosslinked with hemicellulose is decomposed and solubilized giving reactive fragments that in a short time repolymerize to form insoluble products [33, 34]. According to this hypothesis, in the case of the batch system, released lignin compounds are confined inside the reactor and ends up mixed with the solid residue, thus leveling the concentration of ASL.

Differently, in the case of the flow-through process, released lignin compounds are conveyed out of the system before they can react, thus still remaining in solution and leading to accumulation of higher lignin concentration in the hydrolysate.

### Influence of treatment time

The behavior of the two different reaction systems was also compared as a function of the cumulative time of treatment of the solid matrix. This comparison was performed fixing the treatment temperature at 200 °C using two different strategies. In one case, a nominal concentration of biomass in water of 50 g/L was simulated for both systems by fixing properly the cumulative volume of treatment water relative to that of Arundo donax. To achieve this choice, in the case of the flow-through system, the water flow rate had to be decreased from 7.0 to 3.5 mL/min when the cumulative treatment time was increased from 20 to 40 min.

Another comparison can be carried out keeping unchanged the flow rate in the open system. By this choice taking as a reference a nominal concentration of biomass of 100 g/L, corresponding in the open system to a flow rate of 3.5 mL/min with a processing time of 20 min, when the treatment time was increased from 20 to 40 min, the nominal concentration value resulted one half (50 g/L).

When the systems were compared at 50 g/L of nominal loading of biomass in water with 20 min of treatment time (Exp. 2FT and 2MW in Tables 4, 5), lower concentrations and yields of dissolved xylose and arabinose were obtained in the open system with respect to the batch reactor. Conversely, much higher concentration and yield in soluble hemicellulose oligomers were obtained in the FT system compared to the batch one. Since experiments were performed with the same KSF_S_, the higher concentration of oligomers and the lower concentration of C5 sugars in the FT system can be explained by the lower value of log(KSF_L_) that is obtained in this experimental set-up due to the smaller residence time at 200 °C (4.3 min instead of 20 min for the MW-heated system). This result is in agreement with the hypothesis that depolymerization and dissolution of hemicellulose constituting the solid biomass generate the xylo-oligomers that in the liquid phase can undergo hydrolysis with rate increasing with the value of KSF_L_.

**Table 4:** Comparison between microwave-assisted batch and fast heating rate flow-through reaction systems: effect of processing time on the product concentrations

Reaction conditions								Monomer conc.			Oligomer/polymer conc.			Degradation product conc.				
Exp.	T (°C)	t (min)	τ (min)	Q (mL/min)	M_b_/V_w_ (g/L)	Log (KSF_S_)	Log (KSF_L_)	Glu (g/L)	Xyl (g/L)	Arab (g/L)	ASL (g/L)	Olig (Cell) (g/L)	Olig (Hemi) (g/L)	Acet (g/L)	Lev (g/L)	HMF (g/L)	F (g/L)	FA (g/L)
2FT	200	20	4.3	7.0	50	4.25	3.58	0.2	1.8	0.4	3.8	0.6	9.3	0.2	0.0	0.0	0.0	0.0
3FT	200	40	8.6	3.5	50	4.55	3.88	1.3	4.5	0.4	3.1	1.6	4.5	2.3	0.0	0.3	0.4	0.8
4FT	200	20	8.6	3.5	100	4.25	3.88	0.5	6.7	0.8	3.7	1.2	13.5	1.5	0.0	0.0	0.6	0.6
2MW	200	20	–	–	50	4.25	4.25	0.9	4.8	1.6	2.2	1.1	2.3	0.2	0.7	<0.1	0.0	0.0
3MW	200	40	–	–	50	4.55	4.55	1.9	3.9	0.0	4.1	0.0	0.0	0.0	3.0	0.2	0.5	0.0
4MW	200	20	–	–	100	4.25	4.25	1.5	8.8	1.3	3.1	1.4	1.3	0.0	2.6	0.0	0.7	0.0

*τ* average residence time, *Q* water flow rate, *M*_*b*_ */V*_*w*_ biomass to water ratio (w/vol), *KSF*_*S*_ kinetic severity factor for the solid phase, *KSF*_*L*_ kinetic severity factor for the liquid phase, *Glu* glucose, *Xyl* xylose, *Arab* arabinose, *ASL* acid-soluble lignin, *Cell* cellulose, *Hemi* hemicellulose, *Acet* acetic acid, *Lev* levulinic acid, *HMF* hydroxymethilfurfural, *F* furfural, *FA* formic acid

**Table 5:** Comparison between microwave-assisted batch and fast heating rate flow-through reaction systems: effect of processing time on the product yields (%w/w)

Reaction conditions								X_tot_ (%)	Monomer yield			Oligomer/polymer yield			Degradation product yield				
Exp.	T (°C)	t (min)	τ (min)	Q (mL/min)	M_b_/V_w_ (g/L)	Log (KSF_S_)	Log (KSF_L_)		Y_Glu_ (%)	Y_Xyl_ (%)	Y_Arab_ (%)	Y_ASL_ (%)	Y_olig(Cell)_ (%)	Y_olig(Hemi)_ (%)	Y_Acet_ (%)	Y_Lev_ (%)	Y_HMF_ (%)	Y_F_ (%)	Y_FA_ (%)
2FT	200	20	4.3	7.0	50	4.25	3.58	40.3	0.4	4.6	0.9	9.7	1.7	24.0	0.6	0.0	0.0	0.2	0.0
3FT	200	40	8.6	3.5	50	4.55	3.88	44.5	2.7	9.5	0.9	6.5	3.4	9.4	4.9	0.0	0.7	0.9	1.7
4FT	200	20	8.6	3.5	100	4.25	3.88	34.1	0.6	7.9	0.9	4.3	1.4	16.0	1.7	0.0	0.0	0.7	0.6
2MW	200	20	–	–	50	4.25	4.25	34.0	1.8	9.8	3.2	4.5	2.3	4.5	0.5	1.4	<0.1	0.0	0.0
3MW	200	40	–	–	50	4.55	4.55	35.0	3.9	8.0	0.0	8.3	0.0	0.0	0.0	6.0	0.3	1.1	0.0
4MW	200	20	–	–	100	4.25	4.25	30.0	1.5	9.0	1.3	3.2	1.0	1.3	0.0	2.7	0.0	0.7	0.0

*τ* average residence time, *Q* water flow rate, *M*_*b*_ */V*_*w*_ biomass to water ratio (w/vol), *KSF*_*S*_ kinetic severity factor for the solid phase, *KSF*_*L*_ kinetic severity factor for the liquid phase, *X*_*tot*_ total solubilized fraction of the initial biomass calculated by Eq. 8, *Glu* glucose, *Xyl* xylose, *Arab* arabinose, *ASL* acid-soluble lignin, *Cell* cellulose, *Hemi* hemicellulose, *Acet* acetic acid, *Lev* levulinic acid, *HMF* hydroxymethilfurfural, *F* furfural, *FA* formic acid

The cumulative amount of dissolved saccharides and oligosaccharides gives an indication of the apparent rate of biomass solubilization. When these values were calculated in the test performed for 20 min with 50 g/L nominal biomass concentration, a higher value was obtained in the case of the FT system (compare exp. 2FT and 2MW in Table 6). This result is in agreement with the hypothesis that the r.d.s. of biomass liquefaction is the mass transfer of oligomers generated in the solid matrix to the aqueous phase. The rate of this step in the FT system should be higher for the better flow-dynamic regime that induces higher values of mass transfer coefficients.

**Table 6:** Cellulose and hemicellulose extraction in the experiments carried out at different reaction time

Experiment	Cellulose extraction (%)	Hemicellulose extraction (%)	Cellulose/hemicellulose extracted
2FT	6	~100	0.06
3FT	19	88	0.21
4FT	5	90	0.06
2MW	16	55	0.30
3MW	34	28	1.19
4MW	17	38	0.45

For the calculations of the reported values, see the section "Methods"

For the batch process at fixed nominal biomass concentration of 50 g/L, the total fraction of dissolved biomass remained about 35 % independently on the treatment time (Exp. 2MW and 3MW in Table 5). For what concerns the product distribution after 20 min, glucose, xylose, arabinose and oligomers accounted for 21.6 % cumulative yield, the other main component being ASL with 4.5 % w/w. When the treatment time was increased to 40 min (Exp. 3MW in Table 5), cumulative yields in simple sugars decreased to 11.9 %, oligomeric compounds being absent, and furfural, levulinic acid and ASL (1.1, 6.0 and 8.3 %, respectively) were detected. The absence of oligomers can be attributed to the higher process severity.

At similar process conditions, in the FT process, the fraction of solubilized biomass changed little with time (Exp. 2FT and 3FT in Table 5) even if more biomass was dissolved with respect to the closed reactor as total solubilized fractions of 40.3 and 44.5 % were reached after 20 and 40 min, respectively. Also in this case the higher fractions of solubilized biomass must be related to the better mass transfer regime obtained in the semi-continuous system with respect to the batch reactor. According to the initial composition of the adopted Arundo donax, it seems that substantially the whole of the hemicellulose is recovered after 20 min in the FT system, as the total yield in solubilized hemicellulose, estimated considering the cumulative amount of Xyl, Arab, F, Acet, FA and related oligomers, accounts for 30.4 % w/w that corresponds to a fraction of hemicellulose extraction of about 100 % (See Exp. 2FT in Table 6). Formic acid was considered to be derived from hemicellulose because the degradation of glucose from cellulose should lead to the formation of FA and Lev [38, 47] but no Lev was detected by HPLC. In this condition, lignin is also partially extracted (9.7 %), while the majority of cellulose is recalcitrant to solubilization under adopted conditions.

When the treatment time was increased from 20 to 40 min in the MW-assisted batch process (Exp. 2MW and 3MW in Tables 5, 6), the yields in cellulose-derived species (Glu, Lev, FA, oligomers) increased from 5.5 to 9.9 %, while yields in hemicellulose-derived compounds (Xyl, Arab, Acet, F and oligomers) decreased from 18.0 to 9.1 % corresponding to a reduction from 55 to 28 %, respectively, of the extracted fraction. This behavior can be explained by the effect of the higher process severity on the formation of pseudo-lignin from C5 and C6 sugars [48]. It was proposed that pseudo-lignin can be obtained from both xylose and glucose having as key intermediates 3,8-dihydroxy-2-methylchromone and 1,2,4-benzenetriol, respectively [49]. Indeed, Popoff and Theander suggested that the formation from furfural of 3,8-dihydroxy-2-methylchromone was possible already at 96 °C [50], while temperatures higher than 290 °C were necessary to obtain 1,2,4-benzenetriol through HMF [49]. Moreover, experimental data supporting the formation of pseudo-lignin under operative conditions similar to those adopted in this study have been reported by Sipponen et al. [51]. Then, the biasing of selectivity of the process towards cellulose derivatives could be attributed to the faster transformation of C5 monosaccharides in pseudo-lignin when the treatment time and then the process severity was increased [52].

Also in the case of the FT apparatus, when the processing time was increased, it was observed a decrease in the yield of dissolved hemicellulose compounds (compare Exp. 2FT and 3FT with Exp. 2MW and 3MW in Tables 5, 6) that changed from 30.3 to 25.6 % (i.e. from about 100 to 88 % of extracted fraction).

The limited modification of the fractions of solubilized biomass and the reduction in the yield of soluble compounds derived from hemicellulose at higher reaction times for both systems could be another effect of the aforementioned transformation of part of dissolved C5 sugars to furfural that further evolves to form pseudo-lignin. In fact, in the literature, it is reported that the formed pseudo-lignin precipitates on the solid matrix, thus decreasing the final concentration of solubilized compounds and increasing the mass of the solid residue [47, 48]. It seems reasonable to hypothesize that C5 degradation is more relevant in the batch reactor rather than in the FT apparatus where sugars are conveyed out of the reactor by the flow rate having less time to evolve chemically.

The concentration of compounds in the hydrolysate is a key parameter to address its valorization. Indeed, it would be desirable that the hydrothermal treatments led to the formation of liquid solutions with significant concentration of dissolved sugars and oligomers to reduce the energetic cost for concentration.

Batch processing systems are expected to allow for production of more concentrated solutions. On the other hand, experiments performed increasing at 40 min the processing time keeping fixed the nominal concentration of biomass in water at 50 g/L, allowed us to collect from the semi-continuous system a liquor with higher concentration of dissolved sugars and oligomers with respect to that obtained in the MW-heated batch system (compare Exp. 3FT and 3MW in Table 5).

It seems interesting to underline that a different trend was obtained for the concentrations of glucose and glucose—derivatives that were always significantly higher in the batch system (Table 6). This result seems a further confirmation that MW irradiation can induce specific effect on the cellulose matrix promoting its depolymerization as previously mentioned when the effect of temperature was discussed.

The effect of processing time was also studied keeping unchanged the flow rate in the open system at 3.5 mL/min with consequent modification of the nominal concentration of biomass with respect to the total fed water; thus, this ratio changed from 50 to 100 g/L when the treatment time decreased from 40 to 20 min (compare Exp. 4FT and 5FT with 4MW and 5MW in Tables 4, 5). In the MW-heated batch system, the amount of biomass loaded in the reactor was increased from 1 to 2 g when the nominal concentration had to be changed from 50 to 100 g/L.

At the highest investigated biomass concentration and after 20 min of treatment time, the yields in C5 sugars in the hydrolysate collected from the two different experimental apparatuses (Exp. 4FT and 4MW in Tables 4, 5) were more similar among them with respect to those measured at lower nominal concentration of biomass. Moreover, in these conditions, the concentration of hemicellulose-derived oligomers in the hydrolysate from the semi-continuous system was ten times higher than that obtained in the MW-assisted batch reactor in agreement with the hypothesis of faster mass transfer of oligosaccharides generated in the solid matrix to the aqueous phase due to the better flow-dynamic regime of the open system and the lower value of KSF_L_. In these experiments, glucose concentration and the ratio among extracted cellulose and hemicellulose were higher in the MW-heated batch (Exp. 4FT and 4MW in Table 6). Moreover, levulinic acid was only detected in the case of the batch process. Concentrations of furfural of 0.7–0.6 g/L were obtained in both systems (Exp. 4FT and 4MW in Table 4). The absence of difference between the furfural concentrations could be attributed to a faster formation of pseudo-lignin from furaldehyde in the MW-heated system.

According to the collected results using a semi-continuous system to pretreat Arundo donax, it is possible to change the performances of the process by a proper selection of the flow rate that can be adjusted to have simultaneously high fraction of biomass solubilization and limited formation of sugar degradation products with limited penalty in terms of dilution of the hydrolysate with respect to a MW-assisted batch processing system.

### Analysis on the solid residue

**Fig. 1:**
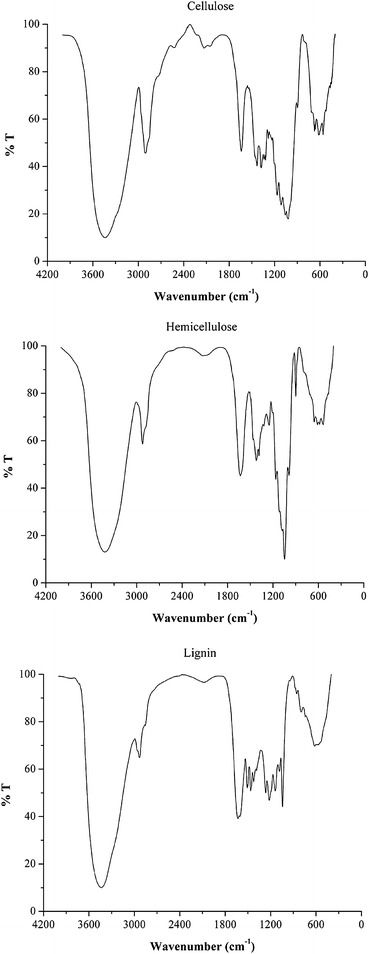
FTIR spectra of cellulose (microcrystalline powder, 99.0 % purity), hemicellulose (xylan from birchwood, 90 % purity) and Kraft lignin (alkali, low sulfonate content) powders used as reference compounds for the main constituents of Arundo donax

**Fig. 2:**
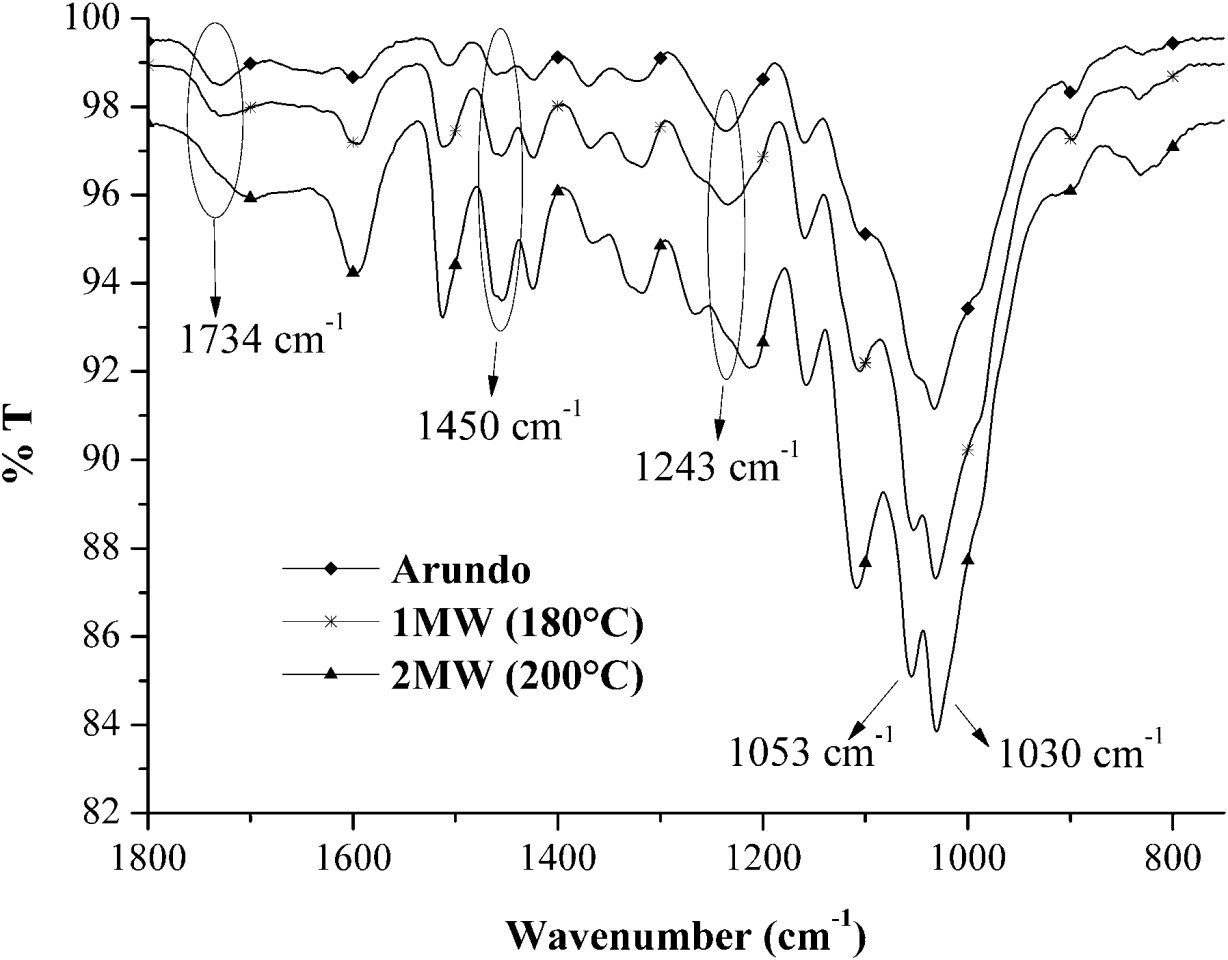
FTIR spectra of the solid residues after the pretreatment of Arundo donax in the MW-assisted batch reactor at two different temperatures; the spectrum of the Arundo donax before the pretreatment is also reported for comparison

**Fig. 3:**
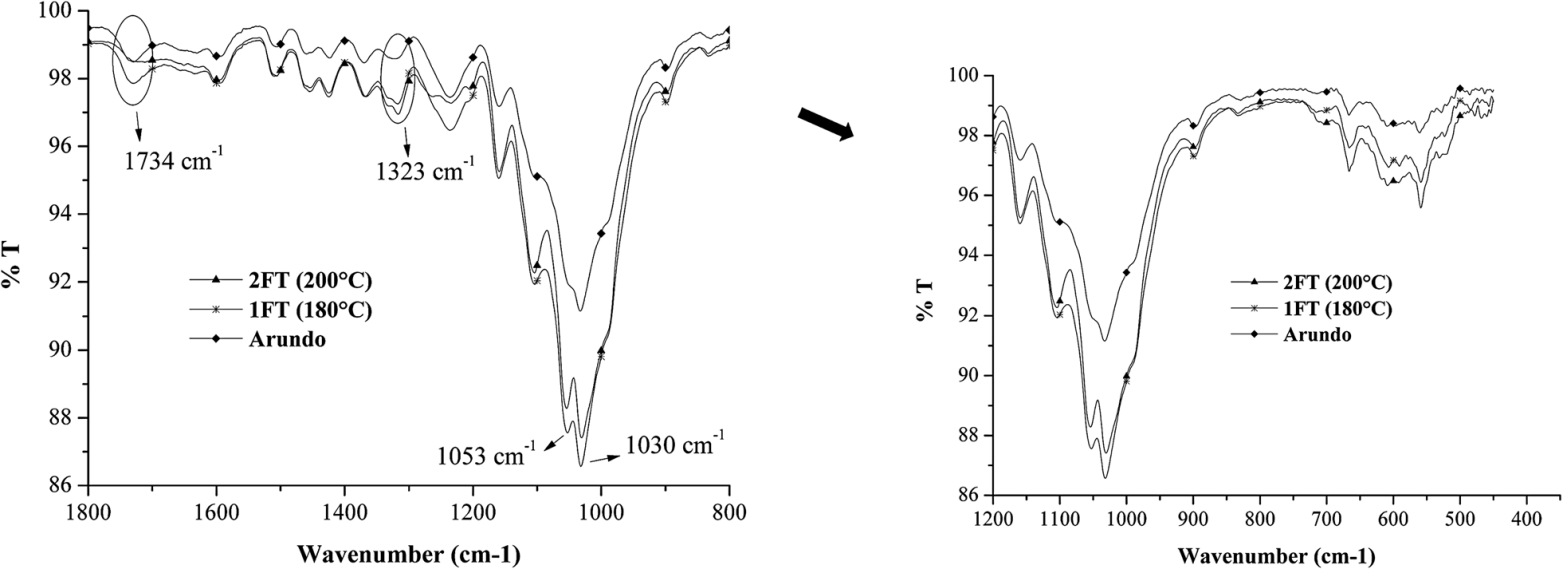
FTIR spectra of the solid residues after the pretreatment of Arundo donax in the flow-through system at two different temperatures; the spectrum of the Arundo donax before the pretreatment is also reported for comparison

Solid residues recovered after pretreatments were characterized by FTIR spectroscopy; this technique offers a useful and quick method to get information on the nature and the composition of both the starting biomass and the recovered solid residues, which can be used to optimize the pretreatment step in a perspective of global valorization of the matrix [53]. Microcrystalline cellulose powder and xylan from birchwood were used as reference compounds for cellulose and hemicellulose respectively, due to their similarity with the different compounds of biopolymers of the starting Arundo donax, as already reported in literature [53]. More difficult was the selection of a model for the lignin that is a very complex polymer of phenyl propane units reciprocally connected by means of different types of bonds. Moreover, commercial lignins are obtained from woody biomass (both hardwoods and softwoods) by processes that can alter the matrix so that differences can be found between the commercial lignin and its native biopolymers incorporated in the starting biomass. For these reasons, it is quite difficult to find a pure lignin matrix that perfectly mimics that of Arundo donax. On the other hand, Kraft lignin has already been found to be a suitable reference compound for the lignin content of solid residues recovered from hydrothermal conversion of Arundo donax in the presence of hydrogen chloride [54]. Considering all these issues, a commercial lignin obtained from Kraft pulping process with NaOH and Na_2_S was chosen as reference compound in this study. Figure 1 shows FTIR of selected reference compounds, whereas spectra of final solid residues collected from batch and FT systems at different temperatures (Exp. 1FT, 2FT and 1MW, 2MW in Tables 1, 2) are presented in Figs. 2, 3, respectively, together with the spectrum of the starting Arundo donax.

The comparison between the starting Arundo donax and the solid samples after MW-assisted treatment at 180 and 200 °C (Exp. 1MW and 2MW in Tables 1, 2) shows a reduction of intensity of the peaks ascribed to the stretching vibration of the functional groups C=O (1734 cm^−1^) and C–O (1243 cm^−1^) of the acetyl ester units in hemicellulose in the treated samples. This attenuation, which becomes more evident when the temperature increases from 180 to 200 °C, is an indication that high percentage of aforementioned ester bonds was cleaved under the adopted hydrolysis conditions [55] and it can be correlated with the fraction of hemicellulose dissolved in the aqueous phase which increased with temperature as previously described. Moreover, in the solid residues, it is possible to observe the increase of the peaks at 1030 and 1053 cm^−1^, indicating the presence of C–O bonds in the C–3 position and the stretching of the C–C and C–O bonds in the C–6 position, respectively, which are typical of cellulose [56]. From HPLC analyses, it was observed that the removal of cellulose for these samples is similar (Exp. 1MW and 2MW in Table 3). A careful inspection of the spectra indicates that the characteristic bands of cellulose and lignin are more evident in the case of the sample obtained from test 2MW than in the sample recovered from test 1MW. For example, the intensity of the peaks at 1450 and 1465 cm^−1^, ascribed, respectively, to the in-plane bending vibrations of the CH_2_ groups characteristic of cellulose and to the asymmetric deformations of C–H bonds typical of lignin, were higher in the sample from 2MW [57]. These observations suggest that the solid residue obtained at the end of test 2MW is richer in cellulose and lignin than sample 1MW.

FTIR spectra of solid samples recovered from the flow-through system studying the effect of temperature are more similar at both investigated temperatures. The removal of hemicellulose (Table 3) was practically quantitative for Exp. 2FT and close to 60 % in the test 1FT in agreement with the higher process severity at 200 °C. The analysis of the FTIR spectra showed that both residues are richer in cellulose and lignin compared to the starting Arundo donax as indicated by the increased intensity of the peaks at 1030 and 1053 cm^−1^ ascribed to cellulose in the treated samples with respect to the corresponding ones observed in the starting biomass.

Moreover, from the comparison between the FTIR spectra of the solid residues recovered from 2FT and 1FT experiments, it was possible to observe that the vibrations of lignin aromatic skeletal at around 1593 cm^−1^, 1458 cm^−1^ and, in particular, at 1323 cm^−1^ were more evident and marked in the solid residue obtained from exp. 2FT due to the quantitative removal of hemicellulose reached in this experiment that caused an enrichment in the concentration of residual lignin.

Finally, the peak at 1734 cm^−1^ of the acetyl ester units in hemicellulose present in the starting Arundo donax decreases in the solid residue of the test at 180 °C (Exp. 1FT), whereas it disappears almost completely in the solid residue at 200 °C (Exp. 2FT), again confirming that this ester bond can be used to mark the dissolution of hemicellulose.

## Conclusions

In this work, the pretreatment of Arundo donax with liquid hot water was investigated in MW-heated batch reactors and in a flow-through system heated by radiant heaters. The same operating conditions were adopted to perform pretreatment experiments of Arundo donax in the two systems to study the effect of the experimental set-up and of various operating parameters, such as temperature, pretreatment time, biomass to water mass to volume ratio and water flow rate, on the hydrolysis of polysaccharides and lignin as well as on the distribution of soluble products in the obtained liquid hydrolysate.

Collected experimental results indicated that the flow-through system can be adopted to obtain effective pretreatment of Arundo donax as complete solubilization of the hemicellulose fraction of the biomass can be obtained. Moreover, by a proper choice of operating conditions, the product distribution in soluble compounds can be addressed towards fermentable sugars. In particular, the use of lower values of KSF_L_ allows one to minimize the concentration of sugar degradation products that are inhibitors of fermentation yeasts.

At the lowest investigated flow rate, more concentrated hydrolysate solutions were recovered from the FT system with respect to the batch one, differently the concentrations of xylose and arabinose in the hydrolysate recovered from the FT system were 6.7 and 0.8 g/L, respectively, while values of 8.8 and 1.3 g/L, respectively, were found in the liquid solution obtained from the batch reactor, thus indicating that the open system can be managed with limited penalty in terms of dilution of the simple sugars. On the other hand, the higher concentration of C6 derivatives obtained in the batch system suggests that microwaves can promote cellulose depolymerization and solubilization, thus allowing a more comprehensive utilization of the biomass in particular if technical solution are found that can match MW heating with the utilization of flow-through processing systems.

## Methods

### Raw materials and analysis procedures

In this work, Arundo donax L. from southern Italy was used as process feedstock. The biomass was first ground as a whole (culms and leaves) in 0.5 mm average size particles. Water and ethanol (HPLC grade) and sulfuric acid (≥95 %) were purchased from Aldrich.

Extractives were separated from the raw biomass through Soxhlet extraction, performed with water and ethanol in sequence according to a procedure reported in the literature [58]. Then, extracted biomass was characterized to determine structural biopolymers (polysaccharides and lignin) [59] and ash [60] amounts, using National Renewable Energy Laboratory (NREL) procedures. In particular, after a two-stage acid hydrolysis treatment, the hydrolysate was recovered by vacuum filtration and analyzed by GC–MS to identify the dissolved sugars. Very small amounts of galactans and mannans were detected in the solutions analyzed by GC–MS and for this reason they were neglected in the calculation of the raw biomass composition. The amounts of monosaccharides, acetic acid and degradation products were quantified by HPLC.

Determination of acid-soluble lignin (ASL) was performed according to NREL procedures analyzing by UV–Vis an aliquot of liquid hydrolysate properly diluted to have absorbance values in the range 0.7–1.0. As suggested by Kaar and Brink, measurements were performed at 205 nm [61].

The amounts of hemicellulose *M*_Hemi_ and cellulose *M*_Cell_ in the raw biomass were estimated using the following equations:

3 $$M_{\text{Hemi}} = 0.88\left( {m_{\text{Xyl}} + m_{\text{Arab}} + 1.56m_{\text{F}} } \right) + 0.98\;m_{\text{AceticAc}}$$

4 $$M_{\text{Cell}} = 0.90(m_{\text{Glu}} + 1.43m_{\text{HMF}} + 1.55\;m_{\text{Lev}} )$$

where *m*_Xyl_, *m*_Arab_, *m*_F_, *m*_AceticAc_, *m*_Glu_, *m*_HMF_ and *m*_Lev_ are the masses of xylose, arabinose, furfural, acetic acid, glucose, hydroxymethylfurfural and levulinic acid, respectively, all quantified by high-performance liquid chromatography. The masses of degradation products HMF, F, Lev were converted in the amount of their starting sugars using conversion factors determined from their stoichiometric equations [38]. When FA molar amount exceeded that of Lev, the excess was added to hemicellulose without any correction factor. This procedure was always used in this study when it was necessary to estimate the amount of starting biopolymer corresponding to the amount of formed degradation products.

The solid residue obtained with the two-stage acid hydrolysis treatment was calcined in a muffle for 24 h at 550 °C for acid-insoluble lignin (Klason lignin) and ash determination. The results of the biomass characterization are reported in Table 7.

**Table 7:** Raw material composition of adopted Arundo donax

Component	Dry matter (% w/w)	Biopolymers (% w/w)
Glucan	36.3 ± 3.0	Cellulose 36.3 ± 3.0
Xylan	28.2 ± 2.0	Hemicellulose 30.0 ± 2.1
Arabinan	1.8 ± 0.1	
Klason lignin	26.3 ± 1.8	Lignin 28.5 ± 2.0
Acid-soluble lignin	2.2 ± 0.2	
Ash	2.4 ± 0.1	–

For each performed test, liquid hydrolysate was analyzed by HPLC according to the NREL procedures [62]. The concentration of dissolved xylose, arabinose and glucose was determined. The concentration of oligomeric polysaccharides in the hydrolysate was quantified by performing an acid hydrolysis of the liquid product in autoclave with 4 % w/w H_2_SO_4_ for 1 h at 121 °C to convert them in monomeric sugars determinable by HPLC, according to NREL procedure [59, 62]. Also the concentrations of molecules derived from the degradation of simple sugars, such as F, HMF, Lev and FA were estimated from chromatographic analyses.

The concentration of soluble lignin (ASL) dissolved in the liquid solution obtained after the autohydrolysis was estimated by UV–Vis spectroscopy with the same procedure previously described to estimate the amount of ASL in the raw biomass.

In all experiments, MW-assisted or in semi-continuous regime, the masses m_i_ of the different compounds previously listed were determined using the equation:

5 $$m_{i} = c_{i} V$$

where *c*_*i*_ are the concentrations [mg/mL] obtained from HPLC or UV–Vis analyses using calibration with pure standards and *V* [mL] is the volume of liquid solution collected at the end of each experiment.

These quantities were used to perform a control mass balance on the processed biomass, according to the following equation:

6 $$M^{ 0} - M^{\text{f}} = \mathop \sum \limits_{i} m_{i}$$

where *M*^0^ is the mass of dry biomass loaded in the reactor and *M*^f^ is the dry mass of the solid residue recovered at the end of the treatment, both of them gravimetrically determined.

Also in these calculations, the masses of HMF, F, Lev and FA were converted in the amount of their starting sugars as previously explained. In all cases, the numerical values of the two members of Eq. 6 differed for less than 10 % of *M*^0^.

The concentrations *c*_*i*_ of the different products in the hydrolysate, previously defined, are reported in the Tables 1 and 4. In the Tables 2 and 5 are reported the product yields *Y*_*i*_ expressed as weight percentage referred to the initial dry biomass (dried at 105 °C up to constant weight) and calculated by the equation:

7 $$Y_{i} = \frac{{m_{i} }}{{M^{0} }}100$$

where both *m*_i_ and *M*^0^ are measured in grams.

The values of cellulose and hemicellulose extraction (%) reported in the Tables 3 and 6 were estimated after the previously mentioned acid hydrolysis of the liquid products in autoclave with 4 % w/w H_2_SO_4_ for 1 h at 121 °C. The total masses of cellulose and hemicellulose solubilized in each test were calculated from the amounts of simple sugars, acetic acid and degradation products with the same method adopted to determine the initial composition of the raw biomass. These values were divided to the initial amount of each biopolymer calculated from the total amount of dry biomass used in each experiment according to the composition reported in Table 7.

The total solubilized fraction of the initial biomass (*X*_tot_) is also reported and was calculated with the following expression from the mass of Arundo donax loaded in the systems and the mass of the solid residue recovered at the end of the treatment, both of them gravimetrically determined on dry basis:

8 $$X_{\text{tot}} \left( \% \right) = 100\frac{{M^{0} - M^{\text{f}} }}{{M^{0} }}$$

For GC–MS analyses, aldoses were silylated and a 6890 N GC System Gas Chromatograph (Agilent Technologies, Palo Alto, CA, USA) equipped with a programmed-temperature vaporizer (PTV) injector and coupled with a 5975 Mass Selective Detector (Agilent Technologies, Palo Alto, CA, USA) single quadrupole mass spectrometer was used. The injector was operated in the constant temperature splitless mode at 250 °C, with a purge time of 0.87 s. The mass spectrometer was operated in the electron impact (EI) positive mode (70 eV). The MS transfer line temperature was 280 °C; the MS ion source temperature was kept at 230 °C; the MS quadrupole temperature was 150 °C. Chromatographic separation was performed on an HP-5MS silica capillary column (J&W Scientific, Agilent Technologies, USA), 30 m × 0.25 mm i.d. (5 % phenyl–95 % methylpolysiloxane). The carrier gas (helium, 99.995 % purity) flow was kept at 1.0 mL/min. The chromatographic oven was kept at 50 °C for 2 min, then from 50 to 190 °C at 5 °C/min, at 190 °C for 20 min, from 190 to 280 °C at 5 °C/min, and 280 °C for 15 min. Both total ion chromatogram (TIC) mode (*m*/*z* 50–650) and selected ion monitoring (SIM) mode were used.

All HPLC analyses were performed with an Agilent 1100 series chromatograph, equipped with a Rezex ROA-Organic-Acid H^+^ column (300 × 7.8 mm, equivalent to Bio-Rad Aminex HPX-87H) and a refractive index detector. 5 mM H_2_SO_4_ in water (HPLC grade) was used as eluent with a flow rate of 0.6 mL/min, the column and detector temperatures were 60 and 40 °C, respectively. A Cary 60 UV–Vis Agilent Spectrophotometer was used for soluble lignin estimation using the Lambert–Beer law on the absorbance peak at 205 nm [61]. Kraft lignin powder (alkali, low sulfonate content) purchased from Sigma-Aldrich was used as reference compound.

FTIR spectra of the initial biomass and of the solid residues recovered at the end of the autohydrolysis treatments were performed using a Perkin Elmer Mod. 2000 spectrophotometer. The acquisition of each spectrum provided 8 scans with a resolution of 4 cm^−1^. Cellulose (microcrystalline powder, 99.0 % purity), hemicellulose (xylan from birchwood, 90 % purity) and Kraft lignin (alkali, low sulfonate content) powders were purchased from Sigma-Aldrich and were used as reference compounds. They were dried at 105 °C in the oven for 12 h and stored in a desiccator before use.

### Experimental set-up and test procedures

LHW experiments have been performed in two experimental apparatuses: a microwave-assisted batch reactor and an in-house assembled semi-continuous (i.e. flow-through) plant.

#### MW-assisted batch reactor

Microwave-assisted batch autohydrolysis experiments of Arundo donax were performed with a commercially available mono-mode microwave reactor (CEM Discover S-class system). In a typical procedure, the selected amount of dry biomass (1 or 2 g depending on the selected value of biomass to water mass to volume ratio) and 20 mL of distilled water were loaded in a 35 mL reactor containing a Teflon-coated magnetic stirring bar. The reactor was capped and heated to the desired temperature under maximum stirring rate. At the end of the reaction time, samples were quenched in an ice bath and filtered with G4 Schott Gouch crucible. The liquid fraction was filtered with 0.2 μm syringe filter prior to HPLC analysis. Each experiment was repeated in duplicate and the reproducibility of this analysis was within 3 %. The solid fraction was washed with water and oven dried at 105 °C to constant weight for further quantification and FTIR characterization.

#### Flow-through layout

**Fig. 4:**
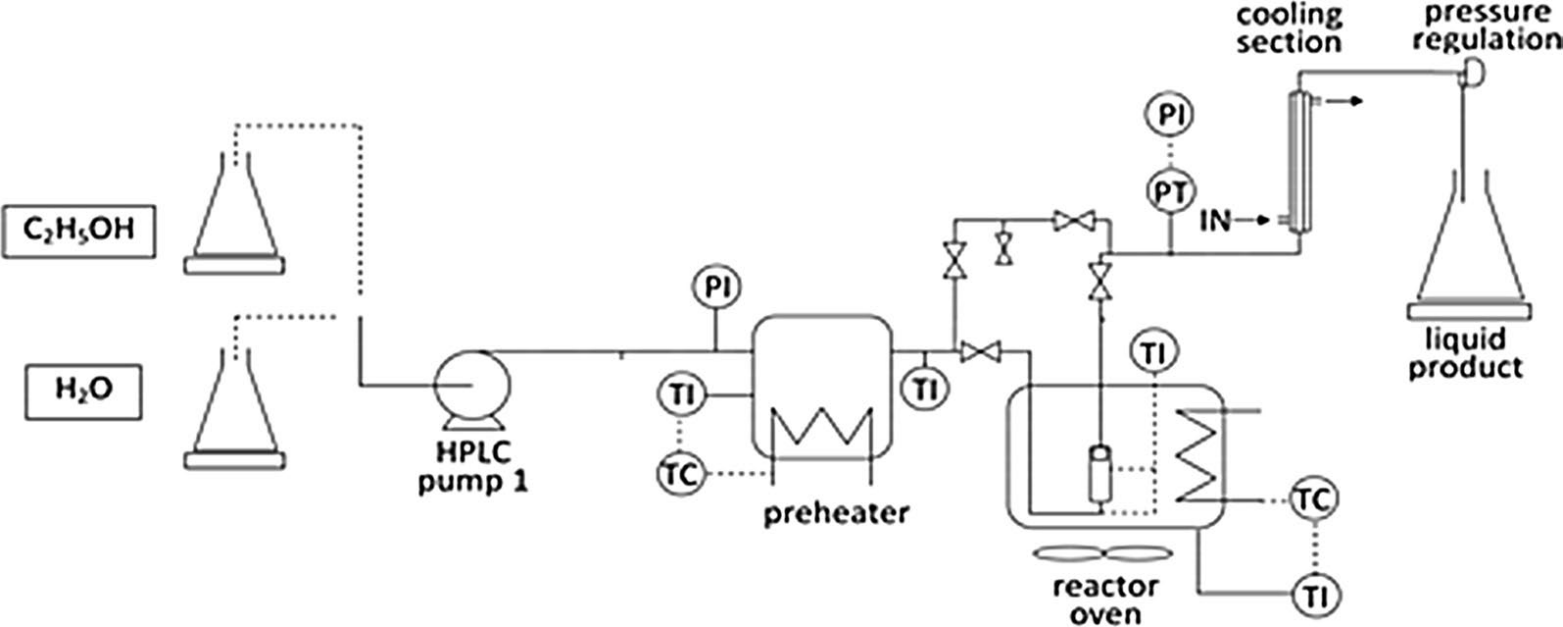
Schematic description of the assembled flow-through apparatus for LHW experiments

In Fig. 4, the lab-scale flow-through plant is schematically described. In a typical experiment, about 7 g of dry comminuted biomass (0.5 mm average particle size) was charged inside the vessel (30 mL volume). The reactor was equipped with a borosilicate glass microfibers filter (Headline) retaining solid particles of 0.1 µm size and with a thermocouple for the measurement of the temperature of the biomass bed during the process. After charging the biomass, the reactor was tightened and the whole system was filled with water at room temperature. An electronically controlled preheating section and a reactor by-pass line made possible a fast heating of the aqueous process stream up to reaction temperature, while keeping it separated from the biomass. During this water preheating step, the reactor initially filled with water and biomass was kept at room temperature. The control of the temperature of the reactor was carried out in a home-made electronically controlled oven by a dual control action: heating provided by a cylindrical ceramic radiant heater as heat source, and air cooling performed using a fan placed at the bottom of the oven. When the water stream reached the set-up temperature, the reactor oven was turned on to heat the biomass/water mixture up to 100 °C (in stopped flow mode). Once 100 °C were reached inside the reactor, the by-pass line was excluded and the hot process water stream from the preheater was fed to the reactor whose outlet valve was opened.

The hot aqueous stream coming out from the reactor was fast cooled to 40 °C in a tubular heat exchanger located soon after the reactor vessel (cooling section in Fig. 4). The system pressure was controlled by a mechanical relief valve located after the tubular heat exchanger, that conveyed the liquid to a collection reservoir.

A vertical lifter allowed moving the reactor oven far from the reactor at the end of the process time thus allowing fast cooling of the vessel by water jet.

The adopted system configuration and the use of radiant heaters allowed short transitory times for both heating and cooling of the reactor at the beginning and at the end of the process, respectively. For example, reactor heating time from 100 °C up to the process temperatures (i.e. transient time for heating) adopted in this study was always less than 5 min.

Once the reactor was cooled down, water at room temperature was used to wash the residual biomass inside the reactor and to recover hydrolysis products dissolved in the hold-up of the apparatus. However, HPLC analysis of the collected washing water revealed amounts of products always lower than 2 % of the total, which were neglected in the calculation of product concentrations and yields.

The solid residue collected after the process was dried at 105 °C up to constant weight for further quantification and FTIR characterization. Some solid particles were also found in the liquid product, probably due to a not perfect fitting of the filter crumble edge with the metal surface of the reactor. These particles were recovered by centrifugation (Thermo Scientific Mod. IEC CL 10) at 3000 rpm for 30 min and filtration over Nylon membranes of 0.2 µm average pore size. The membranes with the retained solids were dried at 105 °C up to constant weight; the amount of the solid was gravimetrically determined and added to the dry amount of the solid recovered from the reactor to perform mass balance calculations.

## Abbreviations

MW: microwave
FT: flow-through
LHW: liquid hot water
KSF_S_: kinetic severity factor for the solid phase
KSF_L_: kinetic severity factor for the liquid phase
Cell: cellulose
Hemi: hemicellulose
Lig: lignin
Glu: glucose
Xyl: xylose
Arab: arabinose
ASL: acid-soluble lignin
Acet: acetic acid
Lev: levulinic acid
HMF: hydroxymethylfurfural
F: furfural
FA: formic acid
